# Heavy-Ion Carbon Radiation Regulates Long Non-Coding RNAs in Cervical Cancer HeLa Cells

**DOI:** 10.7150/jca.30846

**Published:** 2019-08-28

**Authors:** Zhi Yang, Qingying Gu, Ying Wang, Bo Liu, Guangming Zhou, Chunlin Shao, Jing Ruan, Renbing Jia, Shengfang Ge

**Affiliations:** 1 Department of Ophthalmology, Ninth People's Hospital, Shanghai Jiao Tong University School of Medicine, Shanghai 200011, People's Republic of China.; 2 Shanghai Key Laboratory of Orbital Diseases and Ocular Oncology, Shanghai 200011, People's Republic of China.; 3 CAS Key Laboratory of Tissue Microenvironment and Tumor, Shanghai Institute of Nutrition and Health, Shanghai Institutes for Biological Sciences, University of Chinese Academy of Sciences, Chinese Academy of Sciences, Shanghai 200031, People's Republic of China.; 4 Department of Radiation Biology, School of Radiation Medication and Protection, Soochow University, Suzhou 215123, People's Republic of China.; 5 Institute of Radiation Medicine, Fudan University, Shanghai, 200032

**Keywords:** Heavy ion carbon, X-ray, Gene Therapy, LncRNA, Cervical Cancer

## Abstract

Improving the effects of radiotherapy, such as heavy ion radiation, is currently a research priority for oncotherapy. Long non-coding RNAs (lncRNAs) are a subtype of noncoding RNAs involved in the therapeutic response to tumor radiotherapy. However, little is known about the variations in lncRNAs that occur after heavy ion radiation therapy. In this study, we established two kinds of Agilent Human lncRNA arrays and examined the effects of heavy ion radiation and X-ray irradiation on HeLa cells. We compared the differences in lncRNA expression (>=2-fold changes) between cells treated with the two types of radiation and control cells and identified 504 lncRNAs and 285 mRNAs that were differentially expressed. Among these lncRNAs, TCONS-00009910 was the most highly up-regulated lncRNA, while NONHSAT060631 was the most down-regulated lncRNA in both groups. To validate these sequencing data, RT-PCR was performed, and similar findings were obtained. GO and KEGG pathway analyses were employed to probe the potential functions of the affected lncRNAs. Numerous lncRNAs were changed after radiation exposure, showing that they may have important functions in the response to tumour radiotherapy. The present findings may help to elucidate the mechanism by which lncRNAs affect the clinical responses of cancer to radiation and may provide potential diagnostic and therapeutic targets for cancer therapy.

## Introduction

Radiological techniques are widely used in modern medicine. In particular, radiation is the main method used for both diagnosing and treating various cancers 1, 2. Compared to traditional irradiation, the use of heavy ions, such as carbon, helium and silicon, results in linear energy transfer (LET) and a Bragg peak that can notably improve the therapeutic gain factor (TGF) by killing cancer cells that would otherwise be insensitive to radiation. Treating cancer with heavy-ion radiation has many physical, emotional and clinical merits. The major advantage of heavy ion radiation is its inverted dose profile, due to the Bragg peak, which features a sharp longitudinal dose drop at the end of the particle range 3. The unique features of these heavy ions allow for increased dosing within the tumor, which will consequently improve the cure rate. Another advantage of this approach is the high LET. In addition to the radiation dose rate, the quality of the radiation can change the consequences of exposure. For instance, LET radiation causes less damage to the normal cells around the tumor than X-ray irradiation and causes greater damage to the malignant cells by depositing higher energy within the tumor 4, 5. The LET value increases with increasing transversal depth to raise the radiation energy deposition 6. Depending on the LET value, the increased biological efficacy of high LET radiation can also be called the relative biological efficacy (RBE) 7, 8. Numerous clinical experiments have been carried out at the HIRFL-CSR of the Institute of Modern Physics, Chinese Academy of Sciences. In irradiated cancer cell lines, the RBE calculated using the D0 value from survival curves increases with an increase in the LET from 150 keV/μm up to 370 keV/μm 9. Although extensive information about the cellular response to heavy-ion radiation has been provided by radiobiological studies, the variation in long-noncoding RNAs (lncRNAs) induced by LET radiation is not fully understood.

LncRNAs are non-protein-coding transcripts that have sequence lengths of 200 bp and above 10, 11 and represent a subclass of noncoding RNAs (ncRNAs). A growing body of evidence suggests that lncRNAs play import roles in tumorigenesis. Although lncRNAs do not produce proteins, they have the potential to regulate gene expression. The regulation is frequently sequence homology-dependent, and there may be homology to different regions of regulated genes. Moreover, while some noncoding RNAs are conserved, many long noncoding RNAs lack conservation. On the basis of previous studies, lncRNAs have been implicated in diverse biological functions, including gene regulation and genomic imprinting. Identifying the lncRNAs affected by irradiation would improve our understanding of the physiological and biological changes that occur due to radiotherapy.

In the present study, we analyzed the expression patterns of lncRNAs and mRNAs in HeLa cells irradiated with heavy-ion (carbon) radiotherapy and compared these with the corresponding patterns obtained in adjacent X-ray-irradiated and non-irradiated HeLa cells. Using microarray technology, we identified more than 600 unique lncRNAs and mRNAs that were significantly up- or down-regulated after radiation. We verified the changes in many of these differentially expressed lncRNAs with qPCR. To provide a deeper understanding of the biological functions of the differentially expressed lncRNAs, we performed GO and KEGG pathway analyses. By establishing a coding-non-coding gene co-expression network, our results identify numerous lncRNAs that potentially play key roles in the response to radiation exposure. Thus, our findings suggest that the expression of some lncRNAs may play an important role in tumor radiotherapy.

## Materials and Methods

### Cell cultures

HeLa Cells were purchased from Shanghai Cell Bank of Chinese Academy of Sciences. HeLa Cells were seeded at 3×10^5^ cells per well in flat-bottomed 6-well plates. HeLa cells were cultured in DMEM supplemented with 10% fetal bovine serum (FBS) and antibiotics. Cells were grown in a 5% CO_2_ cell culture incubator at 37^◦^C.

### Sample preparation and RNA extraction

Heavy-ion carbon, X-ray and non-radiation irradiate paired HeLa cells. Then all the cells were snap-frozen in liquid nitrogen immediately after radiation and stored at -80˚C until use. According to the manufacture's protocols, we used the Qiagen's miRNeasy Mini Kit (Qiagen, Germany) to extract total RNA from frozen cell pallets, which were then eluted with 60µL of RNase-free water. Total RNA was quantified with the NanoDrop ND-2000 (Thermo Scientific) and the RNA integrity was assessed using Agilent Bioanalyzer 2100 (Agilent Technologies).

### LncRNA and mRNA microarray expression profiling

According to the manufacture's standard protocols, we have got to label the sample, hybridize microarray and wash them in order. Thanks to the Qiagen's miRNeasy Mini Kit, we purified mRNA from total RNA and simultaneously removed the rRNA. Afterwards, each sample was prepared through all of these steps in order: transcribed to double strand cDNA, synthesized into cRNA and labeled with Cyanine-3-CTP. Then, the Human lncRNA array V4.0 (4× 180K, Agilent) could be hybridized onto the prepared labeled cRNAs, also including the global profiling of 78,243 human lncRNAs and 30,215 coding transcripts. Wash the sample to remove the probes that haven't hybridized to cRNAs. Through all the above steps, the sample could be ready to be scanned with the Agilent Scanner G2505C (Agilent Technologies). Genespring (Version 12.5, Agilent Technologies) was employed to finish the basic analysis of the raw data. Feature Extraction software (version 10.7.1.1, Agilent Technologies) was used to analyze array images and extract the raw data. Firstly, we normalized the raw data with the quantile algorithm. Then we filtered the probes with at least 1 condition out of 2 conditions flagged as “P” to analysis with more methods. Fold-change and P value calculated with t-test were chosen as key evaluation indicators to identify differentially expressed lncRNAs and mRNAs. In this project, we define a threshold for up- and down-regulated genes was fold change≥2.0 and p value≤0.05. Finally, all the distinguishable lncRNAs and mRNAs expression patterns among the samples were displayed using the Hierarchical Clustering.

### GO analysis and KEGG analysis

In order to determine the biological roles of these differentially expressed mRNAs, GO analysis and KEGG analysis were performed base on the latest KEGG (Kyoto Encyclopedia of Genes and Genomes) database (http://www.genome.jp/kegg/). We evaluated the significance of the pathway interconnected with the conditions by p value (Hyper geometric-P value). We recommend the threshold of p-value to be 0.05.

### Construction of the co-expression network

Differentially expressed lncRNAs were defined as co-expressed with potentially trans-regulated protein-coding genes, which is beyond 100kb in genomic distance from, or on the other allele of the lncRNAs. MATLAB 2012b (The MathWorks) was used with the hypergeometric cumulative distribution function to construct the lncRNAs-Transcription factors (TFs) network. Cytoscape 3.01 (Agilent and IBS) was used to draw the graph of the lncRNAs-TFs network. We can predict that those lncRNAs may take part in pathways regulated by lncRNAs-Transcription factors when the intersection of these two groups is large enough (FDR < 0.01, under the control of the Benjamini and Hochberg procedure and P < 0.01, calculated by hypergeometric cumulative distribution function).

### RT-PCR

A one-step reaction process was used for quantification reverse transcription (RT) and PCR. Each RT reaction consisted of 1µg RNA, 10.0 µl of 5×QIAGEN OneStep RT-PCR Buffer, 2 µl of dNTP Mix (containing 10mM of each dNTP), 0.6µM of Primer A and B and 2.0µl of QIAGEN OneStep RT-PCR Enzyme Mix (QIAGEN, Germany), in a total volume of 50µl. Reactions were performed in a SimpliAmp Thermal Cycler (Applied Biosystems, ThermoFisher) for 30 min at 50˚C, followed by 30 cycles of denaturation, annealing and extension to produce the right cDNA. Each qPCR reaction consisted of 10.0 µl of Fast SYBR Green Master Mix (2×), 200nM of each forward and reverse primers, 20ng of cDNA and RNase-free water in a total volume of 20µl. Reactions were performed in Applied Biosystems 7500 Fast Real-Time PCR System for 3sec at 95˚C, followed by 30 sec at 60˚C for 40 cycles. All experiments were done in triplicate. At the end of the PCR cycles, melting curve analysis was performed to validate the specific generation of the expected PCR product. Glyceraldehyde-3-phosphate dehydrogenase was performed as control of lncRNAs. All the data were calculated by the method of 2∆Ct.

### Statistical analysis

All the statistical analyses were done with the Statistical Program for Social Sciences (SPSS) 18.0 software (SPSS, Chicago, IL, United States), and were expressed as the mean ± SD or proportions where appropriate. For comparisons, paired t-tests and unpaired t-tests were performed where appropriate. All the graphs was made by GraphPad Prism 5.0 for Microsoft Windows (GraphPad Software, San Diego, CA, United States). P values of 0.05 (two-tailed) were considered statistically significant.

## Results

### mRNAs and lncRNAs aberrantly expressed in HeLa cells after radiation exposure

**Figure 1:**
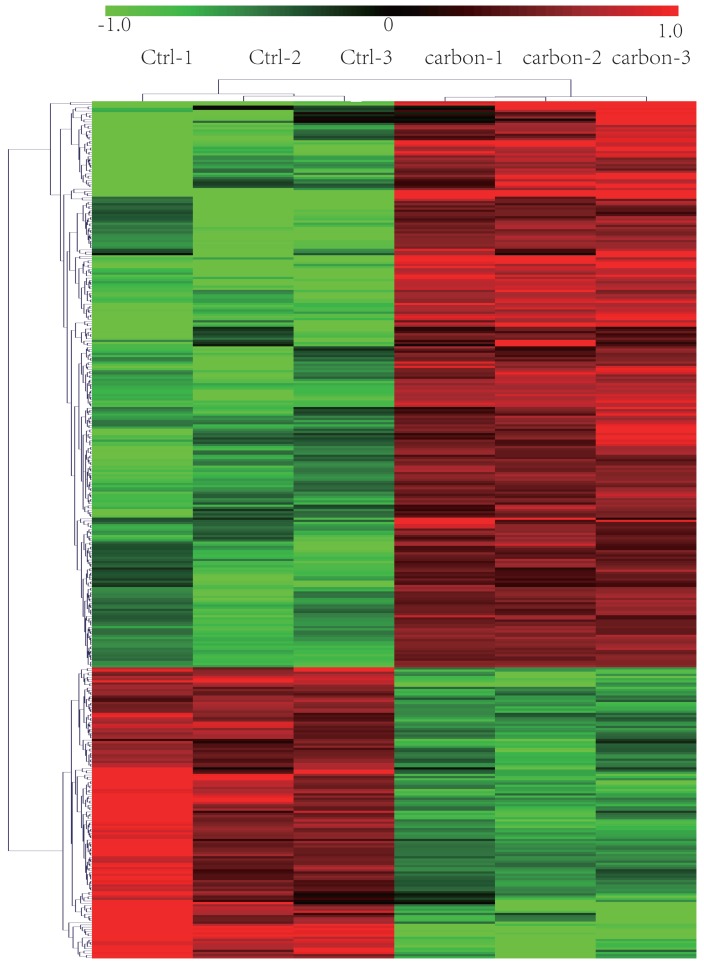
** Heat map and hierarchical clustering of lncRNA profile comparison between the heavy-ion carbon radiation and non-radiation.** Each row represents one lncRNA, and each column represents one cell sample. The relative lncRNA expression is depicted according to the color scale. Red indicates up-regulation; green indicates down regulation. 1.0, 0 and -1.0 are folds changes in the corresponding spectrum, whereas Ctrl represents non-radiation cells and carbon represents radiation.

We sequenced three kinds of cells: heavy-ion (carbon)-irradiated HeLa cells, X-ray-irradiated HeLa cells and normal (control/non-irradiated) HeLa cells. We identified 248 lncRNAs that showed significantly different expression levels (at least a two-fold change) following exposure to heavy-ion carbon irradiation compared with their expression in paired normal cells. Among the differentially expressed lncRNAs, 183 lncRNAs were up-regulated, and 65 were down-regulated. The top 30 lncRNAs that were differentially expressed based on the microarray analysis are shown in **Table 1**. As seen in the table, TCONS_00009910 was the most highly up-regulated lncRNA, with an FC of 13.76. NONHSAT121686 was the most down-regulated lncRNA, with an FC of 5.72. Based on the paired analyses of lncRNAs, 69 down-regulated and 78 up-regulated mRNA transcripts were detected. Among the differentially expressed mRNA transcripts, NM_078467 (CDKN1A) and NM_172239 (REXO1L1) were the most up- and down-regulated, with FC values of 5.97 and 5.35, respectively (shown in **Table 2**). Hierarchical clustering of lncRNAs and mRNAs was performed with the cluster 3.0.2 software. The hierarchical clustering of the expression of 248 lncRNAs and 147 mRNAs, which was based on their centred Pearson correlations, clearly differentiated the HeLa cells exposed to heavy-ion carbon irradiation from normal HeLa cells (**Figure 1**).

### Verify the correctness of the microarray data by qPCR

**Figure 2:**
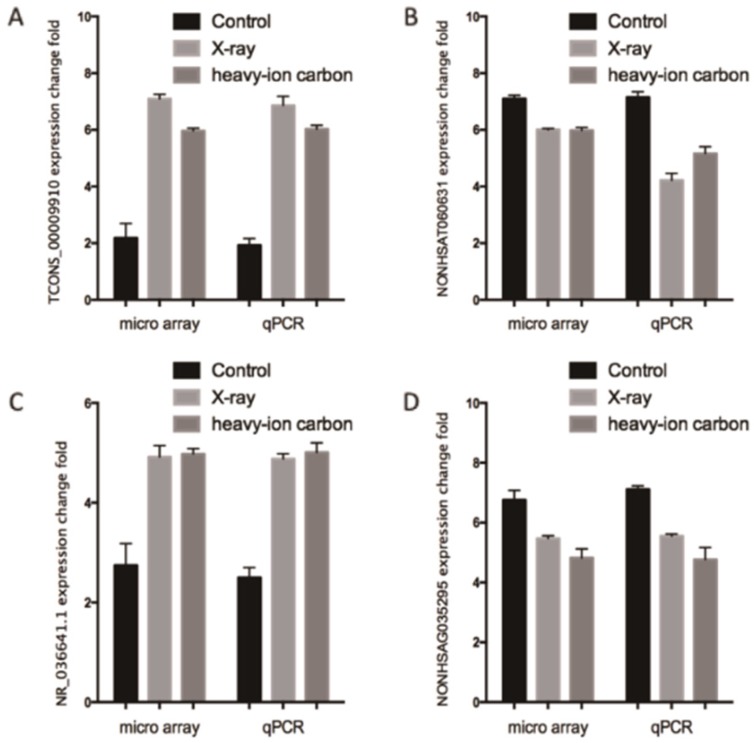
** Comparison between microarray data and qPCR results.** A. TCONS_00009910, B. NONHSAT060631 C.R_036641.1 and D. NONHSAG035295 which were determined to be differentially expressed in radiation samples compared with non-radiation samples in 3 paired samples by microarray was validated by qPCR. The heights of the columns in the chart represent the log-transformed median fold changes in expression for the lncRNA validation; the bars represent standard errors. The validation results of the lncRNAs indicated that the microarray data correlated well with the qPCR results.

We performed high quality microarray experiment according to the assessment results (**Figure S1, Table S1 and Table S2**). Among the lncRNAs transcripts, the most upregulated TCONS_00009910 and downregulated lncRNAs NONHSAT060631 were selected to be verified the correctness of the microarray data with qPCR. Additionally, we choose another two lncRNAs (NR_036641.1 and NONHSAG035295) stochastically for validation the microarray consistency with qPCR. From the results, we found that NONHSAT060631 and NONHSAG035295 were down-regulated and that TCONS_00009910 and NR_036641.1 were up-regulated in the heavy-ion irradiated HeLa cells compared with non-radiation HeLa cells** (Figure 2)**. All these qPCR data are proved to be consistent with the sequenced data.

### KEGG and GO pathway analysis

We referred the analysis method mentioned in the paper 12 in order to predict the function of the lncRNAs. First of all, we calculated the co-expressed mRNAs for each of the differentiated lncRNAs. Besides, a functional enrichment analysis of this set of co-expressed mRNAs was conducted. From the results gotten from upper steps, we can find many enriched functional terms, and then identify them as the predicted functional terms for the given lncRNAs.

**Figure 3:**
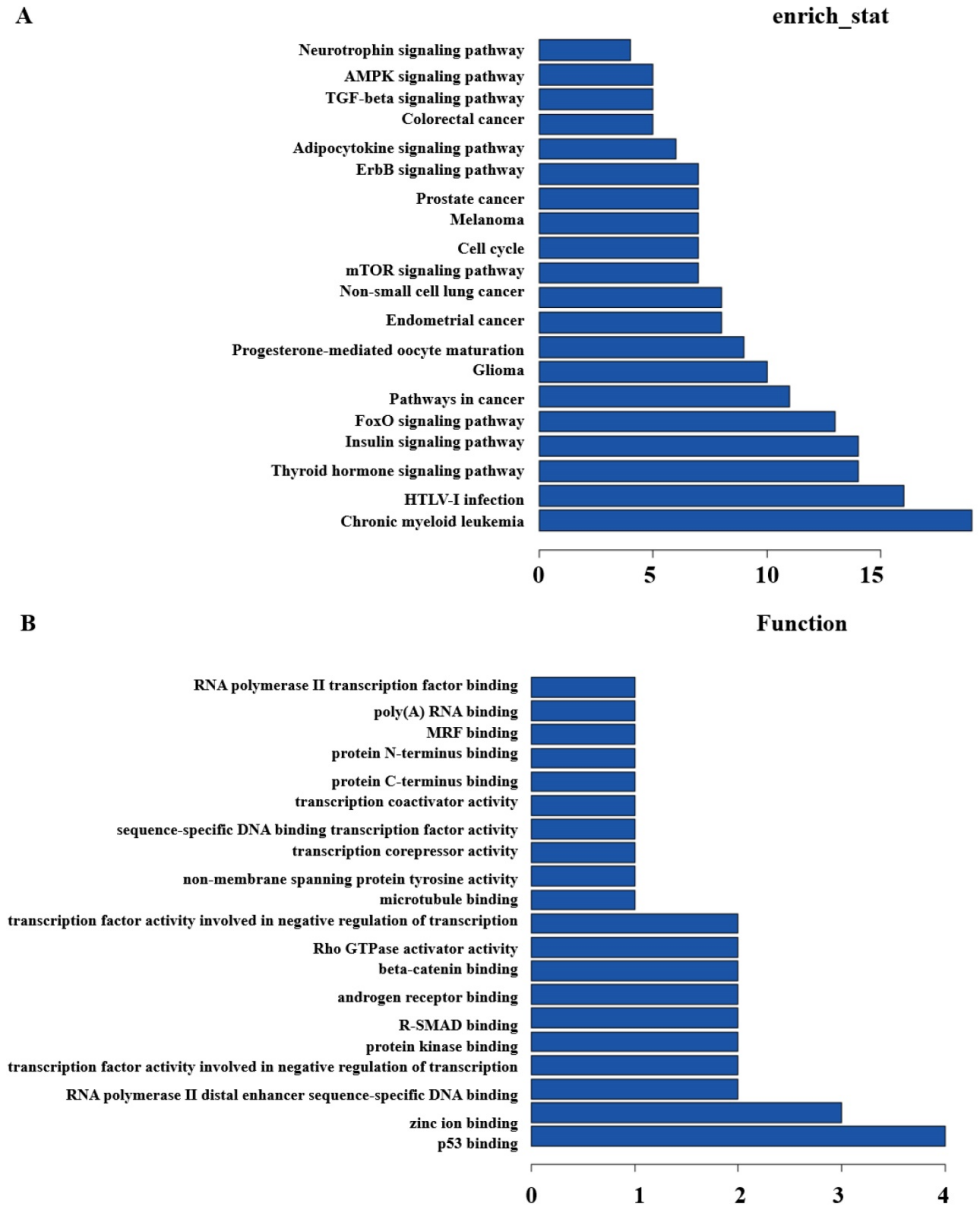
** KEGG and GO Pathway analysis.** (A) KEGG and (B) GO analysis of aberrantly expressed lncRNAs in heavy-ion carbon radiation and X-ray VS control.

Secondly, GO and Pathway analysis with the top200 differentially expressed lncRNAs and mRNAs were run to quest the potential associations between them. KEGG Pathway analysis indicated that several pathways were consistent, including Glioma, Insulin signaling pathway, Thyroid hormone signaling pathway, cell cycle, pathways in cancer, etc. (**Figure 3A**). GO analysis showed that some functional pathways were enriched. Among all of the pathways, p53 binding, RNA polymerase II distal enhancer sequence-specific DNA binding, protein kinase binding, Rho GTPase activator activity, transcription coactivator and corepressor activity were the most closely associated with heavy-ion carbon irradiated cells (**Figure 3B**).

### LncRNAs and mRNAs that are dysregulated both in heavy-ion carbon and X-ray irradiated HeLa cells

**Figure 4:**
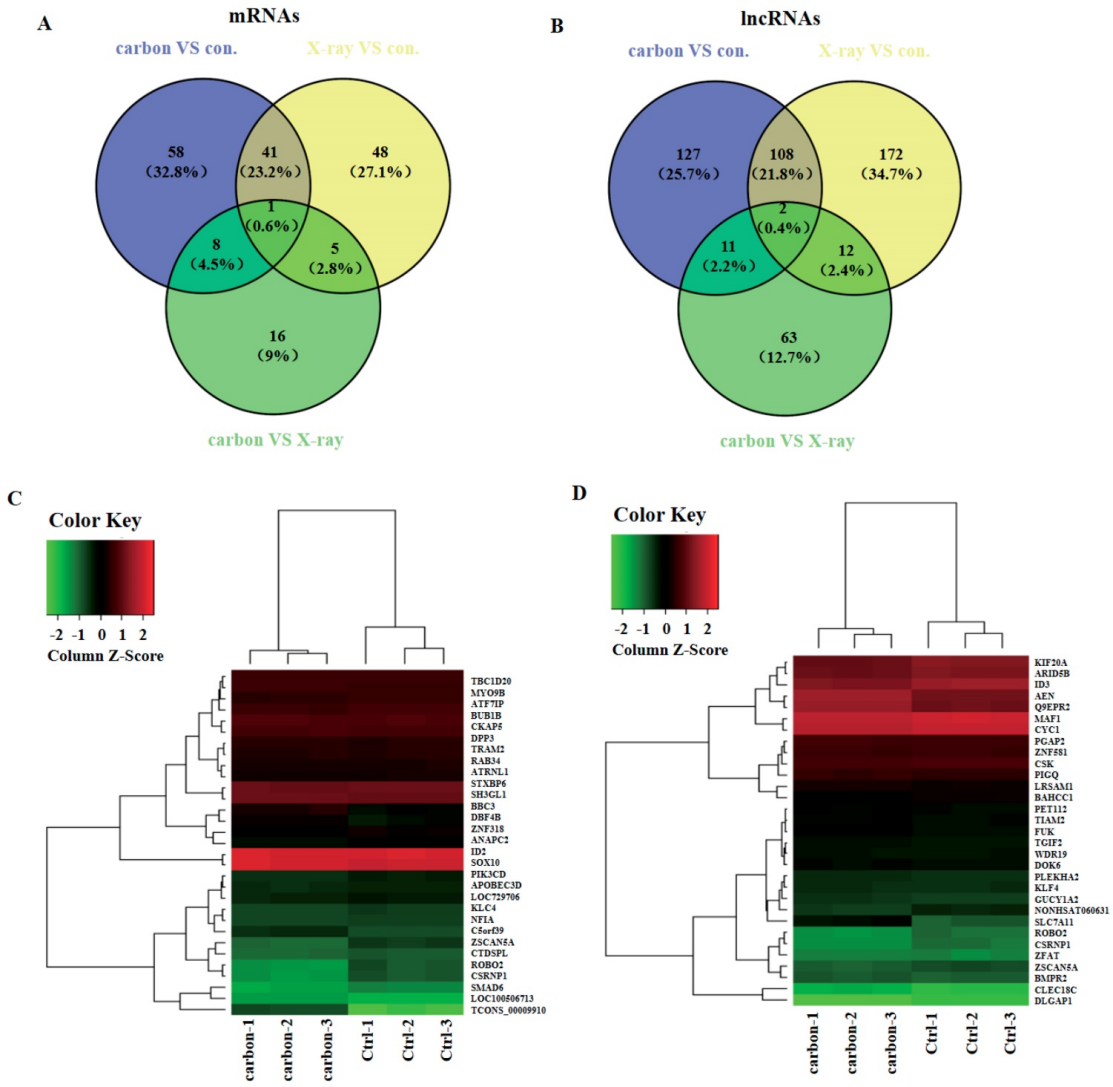
** Venn diagram and heat map**. (A) Venn diagram of mRNAs and (B) lncRNAs from heavy-ion carbon radiation cells, X-ray radiation VS non-radiation. (C) Heat map and hierarchical clustering of genes profile according to TCONS_00009910 and (D) NONHSAT060631.

In order to explore which lncRNAs and mRNAs play an important role in radiation treatment, three paired samples were sequenced including heavy-ion carbon radiation, X-ray radiation and no radiation. LncRNAs and mRNAs profiling detected 42 mRNAs and 110 lncRNAs that were significant dysregulated both in heavy-ion carbon and X-ray irradiated HeLa cells (**Figure 4A-B**). Among the 110 lncRNAs transcripts, TCONS_00009910 was the most up-regulated, with an FC of 13.76, whereas NONHSAT060631 was the most down-regulated, with an FC of 2.16.

Then we constructed a co-expression network based on the correlation analysis between the lncRNAs and mRNAs. LncRNAs and mRNAs with Pearson's correlation coefficients of no less than 0.99 were used to construct the network. The mRNAs that co-expressed with these lncRNAs and those are regulated by certain Transcription factors (TFs) were compared in order to find those lncRNAs, which possibly have trans-regulating functions. Finally, data suggest that ROBO2, CSRNP1, SMAD6 may have important relationship with the most upregulated lncRNA TCONS_00009910 (**Figure 4C**). Furthermore, using the same criteria, SLC7A11, AEN, Q9EPR2 may play central roles in lncRNAs process (**Figure 4D**).

## Discussion

Few studies have focused on what's the function of lncRNAs in radiation treatment. Gezer U et al. irradiated HeLa and MCF-7 with γ-ray and then evaluated the function of several lncRNAs like HOTAIR, MALAT1, TUG1, lincRNA-p21, GAS5, MEG3, PANDA, UCA1, ANRIL and CCND1, etc. in the process of cell death induced by DNA damage. The results showed that the expression level of lncRNAs varied as cell type changes. In the process of radiation induced cell death. Generally speaking, HOTAIR and MALAT1 were down-regulated, lincRNA-p21, GAS5, MEG3, ANRIL and CCND1 were up-regulated, while TUG1, UCA1 and PANDA didn't effect on the process 13. One professor preliminarily screened the lncRNAs sensitive to colon cancer cells. After screening the microarray of lncRNAs, 268 lncRNAs were selected with positive expression and closely related to CCND1 that encodes the protein cyclin D1. Further analyses revealed that lncRNAs may combine with CCND1 through transcription complex to regulate the expression of cyclin D1. In consequence, colon cancer cells become more sensitive to radiation 14. Radiotherapy of nasopharynx cancer patients may induce the observably alteration of lncRNAs especially lncRNA AK294004, a negative regulator of CCND1, which participate in the process of cancer resistance 15. All these results indicated that lncRNAs play an important role during the process of tumor radiotherapy. The expression of certain lncRNAs changed markedly and may also take part in the regulation of key signal pathways. Increased understanding of the role of the potential lncRNAs in the process of radiotherapy of cervical cancer could help us find more potential targets during the healing process.

In my project, we sequenced three paired microarray of HeLa irradiated with different radiation, and luckily we found lots of quantitatively and significant differentially expressed lncRNAs and mRNAs according to control. Radiations we used like heavy-ion carbon and X-ray are commonly used in clinical radiotherapy. So with the invitro simulative radiotherapy and abundant and varied probes, 504 lncRNAs and 285 mRNAs were determined significantly and measurable differential expression. What's more, the truthfulness and the accuracy of the data from the microarrays were validated with qRT-PCR, in consequence, the results were consistent with the sequenced data. Some specific expressed lncRNAs in heavy-ion carbon irradiated HeLa cells rather than X-ray or control, and some other specific ones both in heavy-ion carbon and X-ray were analyzed emphatically. However, we know little about most of the differentially expressed lncRNAs corresponded to novel transcripts 16, 17. To solve this problem, we run GO analysis and KEGG pathway annotation in the lncRNA target gene pool. GO analysis found that the more genes corresponding to up-regulated lncRNAs than corresponding to down-regulated lncRNAs. So those pathways may have important function during the process of radiotherapy. Increasing understanding of the role of these lncRNAs and pathways may mediate momentously and meaningfully radiotherapy process.

However, the research of lncRNAs is just starting out according to encoding gene and microRNA. And it remains to be seen how the lncRNAs function as in the body. So far, the studies of lncRNAs mostly focused on their biological functions, their patterns of the adjustment of protein transcription and the activity of regulatory proteins. All those studies open a wide field for researchers to dive. Actually, lncRNAs played an important role during the process of tumorigenesis. The expression level of some certain lncRNAs could change a lot in tumor cells, so the change could be the potential marker for cancer diagnoses and medicine targets. Pro Song et al. found that lncRNA MALAT-1 can interact with protein hPSF to remove the protein's inhibition of certain cancers 18. Certain professor discovered a specific up-regulated lncRNA HEIH through lncRNAs expression profiles. Results showed that lncRNA HEIH could combine with EZH2 to recruit PRC2, and then inhibit the target genes 19. Meanwhile, researchers from MIT, Howard and Stanford University found lncRNAs regulated by p53. Results showed that p53 could improve the expression level of lncRNA-p21. And then lncRNA-p21 combined with protein hnRNP-R to further regulate the expression level of his target genes. Furthermore, the results all showed that lncRNAs could participate in the regulation of the activity of protein by forming certain the secondary structure 20, even though the function of lncRNAs in the tumor cells after radiotherapy has been poorly understood. As more data become available, it will have some better explanations.

To summarize, we analyzed the expression profiles of differential lncRNAs and mRNA of three paired HeLa cells irradiated by heavy-ion carbon, X-ray and non-radiation. What's more, the target genes of most differential lncRNAs were predicted to guess their role in regulation of protein kinase and cell death. Further investigation of the lncRNAs discovered in this project will focus on their biological functions and their expression level in normal cells and tumor. Our study provides useful information to improve our understanding of the physiological and biological change after radiotherapy.

## Supplementary Material

Supplementary figure and tables.

## Figures and Tables

**Table 1:** Top 30 differently expressed lncRNA in microarray for three pairs of HeLa irradiated by heavy-ion carbon and adjacent non-radiation cells

Target ID	p	FC (abs)	Regulation	Carbon1	Carbon2	Carbon3	Con1	Con2	Con3	ncRNA_Accession	Chr
TCONS_00009910	0.01	13.76	up	6.07	5.92	5.92	1.67	2.69	2.21	TCONS_00009910	chr5
ENST00000605049	0.03	10.84	up	4.79	5.24	5.40	1.02	1.00	3.09	ENST00000605049	chr8
ENST00000512067	0.01	8.48	up	5.38	5.56	5.49	2.73	2.06	2.39	ENST00000512067	chr5
NONHSAG043674	0.01	7.11	up	10.60	10.60	10.61	8.32	7.55	7.47	NONHSAG043674	chr6
NONHSAT121686	0.01	5.72	down	3.23	1.86	1.44	5.68	4.07	4.33	NONHSAT121686	chr7
NONHSAT130623	0.02	5.60	down	3.68	3.60	3.90	6.81	5.82	6.01	NONHSAT130623	chr9
TCONS_00005565	0.04	5.04	up	4.59	3.86	4.74	3.04	1.63	1.52	TCONS_00005565	chr3
NONHSAG051970	0.01	5.02	down	4.50	4.54	4.51	7.15	6.61	6.77	NONHSAG051970	chr9
NONHSAT025343	0.01	4.86	up	3.85	4.10	3.93	1.59	2.16	1.28	NONHSAT025343	chr12
NR_036641.1	0.01	4.70	up	4.95	5.10	4.89	2.24	3.03	2.97	NR_036641.1	chr5
NONHSAT107261	0.02	4.65	up	3.95	4.08	3.91	1.03	2.22	2.04	NONHSAT107261	chr6
FR239613	0.01	4.53	down	4.43	3.45	3.76	6.18	6.08	5.92	FR239613	chr10
NONHSAT029249	0.01	4.53	down	3.12	3.61	3.74	5.78	5.70	5.53	NONHSAT029249	chr12
NONHSAT133169	0.01	4.52	up	6.19	6.08	6.34	4.31	3.93	3.84	NONHSAT133169	chr9
NONHSAT030250	0.00	4.47	up	3.63	3.46	3.72	1.45	1.11	1.77	NONHSAT030250	chr12
NONHSAG034228	0.04	4.36	up	4.18	4.23	4.41	2.05	1.38	3.03	NONHSAG034228	chr22
NONHSAT100801	0.02	4.33	up	4.66	4.85	4.47	1.98	2.77	2.88	NONHSAT100801	chr5
NONHSAT136766	0.00	4.31	up	5.16	5.20	4.32	2.91	3.06	2.39	NONHSAT136766	chrX
NONHSAT076513	0.02	4.21	down	4.12	4.05	3.25	6.31	5.62	5.72	NONHSAT076513	chr2
NONHSAG009130	0.01	4.16	up	3.35	3.81	3.87	0.98	2.11	1.77	NONHSAG009130	chr11
NONHSAT101069	0.02	4.09	up	7.14	7.13	7.47	4.71	5.70	5.24	NONHSAT101069	chr5
XR_246234.1	0.02	4.04	up	4.84	4.83	4.93	2.32	3.18	3.06	XR_246234.1	chr6
XR_243289.1	0.04	3.98	up	3.96	3.60	3.71	1.14	2.26	1.89	XR_243289.1	chr7
NONHSAT005197	0.04	3.96	up	6.28	6.00	5.98	5.05	3.82	3.43	NONHSAT005197	chr1
TCONS_l2_00011998	0.03	3.94	down	2.15	2.27	2.00	4.87	3.90	3.59	TCONS_l2_00011998	chr18
NONHSAT047555	0.03	3.90	up	3.56	3.51	3.70	0.99	1.71	2.18	NONHSAT047555	chr15
NONHSAT104286	0.02	3.87	up	3.20	3.30	3.56	1.00	1.88	1.33	NONHSAT104286	chr5
NONHSAG035295	0.00	3.83	down	5.17	4.70	4.60	7.12	6.65	6.50	NONHSAG035295	chr3
ENST00000436942	0.00	3.76	up	5.04	5.01	5.19	3.06	3.21	3.23	ENST00000436942	chr10
NONHSAG002901	0.02	3.74	up	3.92	4.25	4.54	1.62	2.22	3.15	NONHSAG002901	chr1

**Table 2:** Top 30 differently expressed mRNA in microarray for three pairs of HeLa irradiated by heavy-ion carbon and adjacent non-radiation cells

GenbankAccession	p	FC (abs)	Regulation	Carbon1	Carbon2	Carbon3	Con1	Con2	Con3	Target ID	Chr
NM_078467	0.01	5.97	up	12.85	12.89	12.66	10.70	9.87	10.10	CDKN1A	chr6
NM_172239	0.04	5.35	down	2.16	2.85	2.74	5.39	4.38	5.23	REXO1L1	chr8
NM_018328	0.00	5.20	down	2.39	2.20	1.90	4.91	4.45	4.27	MBD5	chr2
NM_001029884	0.01	4.31	up	3.28	3.72	3.34	1.40	1.17	1.45	PLEKHG1	chr6
NM_000214	0.00	4.29	up	5.94	5.88	5.67	3.92	3.49	3.78	JAG1	chr20
DA992326	0.01	4.25	down	2.90	1.81	3.30	5.22	4.08	4.97		chr10
NM_152570	0.01	4.15	down	4.27	4.42	4.37	6.67	6.26	6.28	LINGO2	chr9
NM_001010848	0.02	3.99	down	3.57	3.49	4.07	6.01	5.52	5.59	NRG3	chr10
AL833126	0.01	3.89	up	3.08	3.42	3.31	1.19	1.70	1.02	FAM178A	chr10
NM_014850	0.03	3.88	down	2.90	1.54	2.85	4.51	4.19	4.46	SRGAP3	chr3
NM_001964	0.05	3.73	down	2.84	2.85	3.13	5.28	5.07	4.17	EGR1	chr5
NM_003633	0.01	3.71	up	9.63	9.72	9.67	8.18	7.58	7.58	ENC1	chr5
NM_001010848	0.00	3.54	down	6.58	6.27	6.63	8.34	8.29	8.31	NRG3	chr10
NM_031422	0.00	3.53	down	3.79	3.42	3.96	5.74	5.33	5.56	CHST9	chr18
NM_152361	0.03	3.45	up	4.38	3.68	3.69	1.94	2.21	2.25	EID2B	chr19
NM_001190468	0.01	3.42	up	4.40	4.37	4.44	2.95	2.49	2.46	GDNF	chr5
NM_000514	0.01	3.40	up	6.71	6.49	6.67	5.27	4.68	4.63	GDNF	chr5
NM_021069	0.01	3.37	down	3.88	3.48	3.65	5.37	5.47	5.43	SORBS2	chr4
NM_033026	0.01	3.34	down	2.67	2.38	2.85	4.46	4.30	4.34	PCLO	chr7
NM_002392	0.00	3.25	up	7.53	7.68	7.58	5.67	6.04	5.97	MDM2	chr12
XM_003403733	0.01	3.23	up	3.66	3.37	3.46	1.93	2.00	1.49		chr20
NM_002228	0.02	3.21	down	3.83	3.49	3.56	5.70	5.52	4.72	JUN	chr1
AK129975	0.01	3.20	up	4.37	4.01	4.33	2.39	2.38	2.90		chr5
NM_002392	0.01	3.19	up	9.74	9.84	9.65	8.24	7.92	8.06	MDM2	chr12
AY338954	0.01	3.18	up	7.45	7.50	7.45	5.55	5.83	6.02		chr21
NM_004864	0.00	3.17	up	8.00	8.11	8.00	6.48	6.43	6.19	GDF15	chr19
XM_003119816	0.04	3.14	up	4.34	4.73	5.88	3.37	2.91	3.73	LOC100509860	chr20
NM_006763	0.00	3.08	up	10.11	10.12	10.20	8.71	8.35	8.50	BTG2	chr1
NM_002392	0.01	3.00	up	6.75	6.78	6.80	5.15	5.40	5.01	MDM2	chr12
NM_001554	0.02	3.00	down	9.24	8.68	8.46	7.23	7.18	7.22	CYR61	chr1
