# Supplementary material for: Heavy-Ion Carbon Radiation Regulates Long Non-Coding RNAs in Cervical Cancer HeLa Cells
Source: J Cancer. 2019 Aug 28;10(21):5022–30. doi: 10.7150/jca.30846 (PMC6775614; doi:10.7150/jca.30846)
Supplement: Supplementary file 1 — Supplementary figure and tables. [file jcav10p5022s1.pdf]

Supplementary Materials

Table S1.RNA Sample Quality Assessment

Sample ID	Concentration (μg/μl)	A260/280	A260/230	Volume (μl)	Total (μg)	28S/18S	RIN	Rating
CTRL-1	0.1411	1.94	0.76	15	2	1.5	9.6	A
CTRL-2	0.1585	1.90	0.63	15	2	1.4	9.9	A
CTRL-3	0.1315	1.89	0.55	15	2	1.5	10.0	A
carbon-1	0.1602	1.93	1.06	15	2	1.5	9.9	A
carbon-2	0.1468	1.98	1.04	15	2	1.6	9.7	A
carbon-3	0.1516	1.96	1.27	15	2	1.5	9.9	A
x-rays-1	0.1624	1.91	1.05	15	2	1.7	9.8	A
x-rays-2	0.1435	1.98	1.08	15	2	1.6	9.7	A
x-rays-3	0.1447	1.95	1.09	15	2	1.6	9.6	A

Table S2. Assessment of Agilent lncRNA Array Experiment

Sample ID	Microarray Number	Fluorescent labeling	CV（%）	Result
CTRL-1	10017-4	cy3	6.42	success
CTRL-2	10018-1	cy3	8.37	success
CTRL-3	10018-2	cy3	8.65	success
carbon-1	10018-3	cy3	8.94	success
carbon-2	10018-4	cy3	8.85	success
carbon-3	10019-1	cy3	8.04	success
x-rays-1	10019-2	cy3	7.64	success
x-rays-2	10019-3	cy3	7.89	success
x-rays-3	10019-4	cy3	7.91	success

A

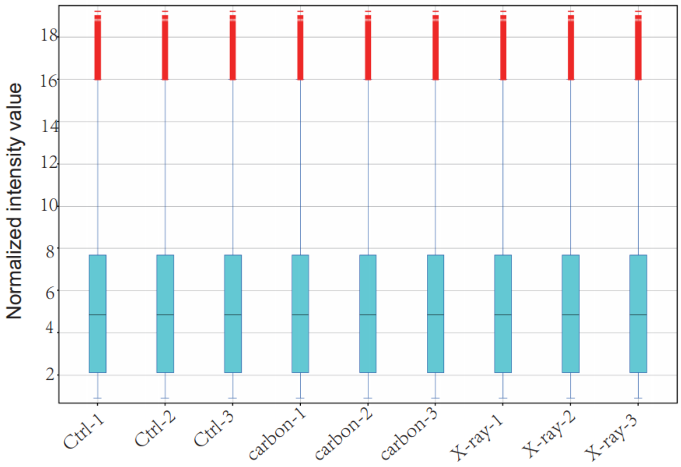

B

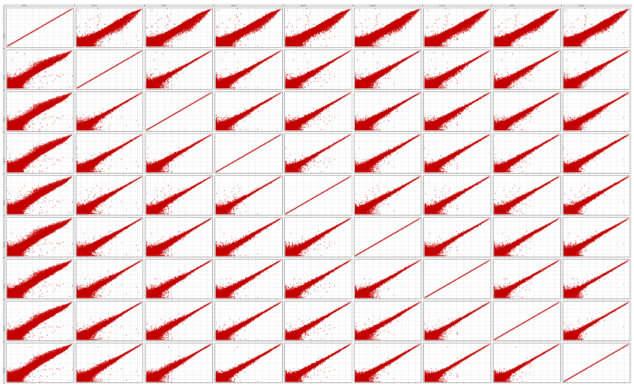

C

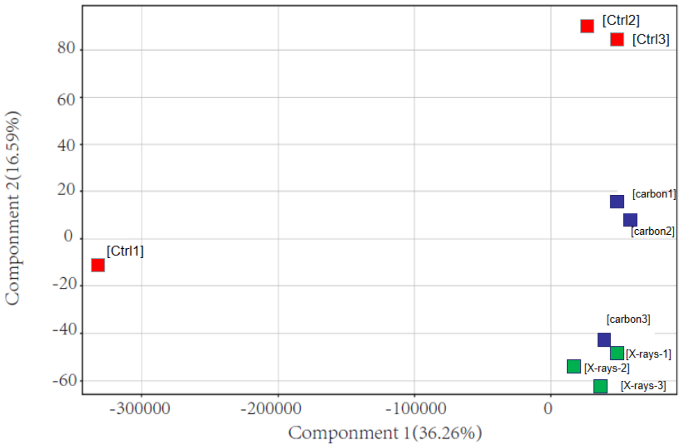

Figure S1. Assessment of lncRNA microarray project. A. Box-whisker Plot. The data has good symmetry and degree of dispersion. B. Scatter plot. The data is used to evaluate the overall distribution trend of the two sets of data. C.PCA. The samples in the same group are concentrated in two-dimensional space, indicating that these genes are representative and biologically repeatable.
